# *catena*-Poly[[(2,9-dimethyl-1,10-phenanthroline-κ^2^ *N*,*N*′)lead(II)]-di-μ-bromido]

**DOI:** 10.1107/S160053681201940X

**Published:** 2012-05-05

**Authors:** Behrous Sabour, Ezzatollah Najafi, Mostafa M. Amini, Seik Weng Ng

**Affiliations:** a Department of Chemistry, General Campus, Shahid Beheshti University, Tehran 1983963113, Iran; b Department of Chemistry, University of Malaya, 50603 Kuala Lumpur, Malaysia; c Chemistry Department, Faculty of Science, King Abdulaziz University, PO Box 80203 Jeddah, Saudi Arabia

## Abstract

In the title compound, [PbBr_2_(C_14_H_12_N_2_)]_*n*_, the Pb^II^ atom lies on a twofold rotation axis. The *N*-heterocycle-chelated Pb^II^ atom exists in a distorted octa­hedral geometry owing to two long Pb⋯Br inter­actions [2.9562 (5) and 3.2594 (5) Å]. These result in a zigzag chain running along the *c* axis. The lone pair is stereochemically inactive.

## Related literature

For the lead(II) bromide–1,10-phenanthroline homolog, see: Bowmaker *et al.* (1996 ▶).
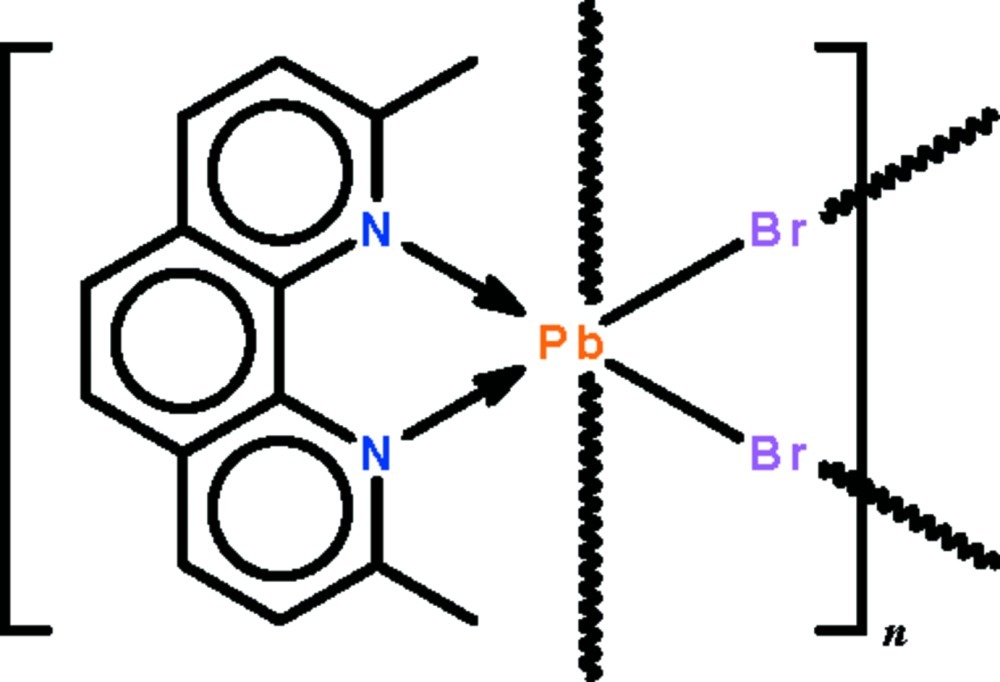


## Experimental

### 

#### Crystal data

[PbBr_2_(C_14_H_12_N_2_)]
*M* *_r_* = 575.27
Monoclinic, 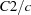

*a* = 18.3852 (13) Å
*b* = 11.8312 (5) Å
*c* = 7.4609 (5) Å
β = 112.346 (8)°
*V* = 1501.02 (16) Å^3^
*Z* = 4
Mo *K*α radiation
μ = 16.55 mm^−1^
*T* = 100 K
0.15 × 0.15 × 0.05 mm

#### Data collection

Agilent SuperNova Dual diffractometer with an Atlas detector
Absorption correction: multi-scan (*CrysAlis PRO*; Agilent, 2012 ▶) *T*_min_ = 0.190, *T*_max_ = 0.492
4947 measured reflections
1734 independent reflections
1620 reflections with *I* > 2σ(*I*)
*R*_int_ = 0.032

#### Refinement

*R*[*F* ^2^ > 2σ(*F* ^2^)] = 0.026
*wR*(*F* ^2^) = 0.058
*S* = 1.01
1734 reflections
88 parameters
H-atom parameters constrained
Δρ_max_ = 1.43 e Å^−3^
Δρ_min_ = −1.45 e Å^−3^

### 

Data collection: *CrysAlis PRO* (Agilent, 2012 ▶); cell refinement: *CrysAlis PRO*; data reduction: *CrysAlis PRO*; program(s) used to solve structure: *SHELXS97* (Sheldrick, 2008 ▶); program(s) used to refine structure: *SHELXL97* (Sheldrick, 2008 ▶); molecular graphics: *X-SEED* (Barbour, 2001 ▶); software used to prepare material for publication: *publCIF* (Westrip, 2010 ▶).

## Supplementary Material

Crystal structure: contains datablock(s) global, I. DOI: 10.1107/S160053681201940X/bt5905sup1.cif

Structure factors: contains datablock(s) I. DOI: 10.1107/S160053681201940X/bt5905Isup2.hkl

Additional supplementary materials:  crystallographic information; 3D view; checkCIF report

## Figures and Tables

**Fig. 1.**
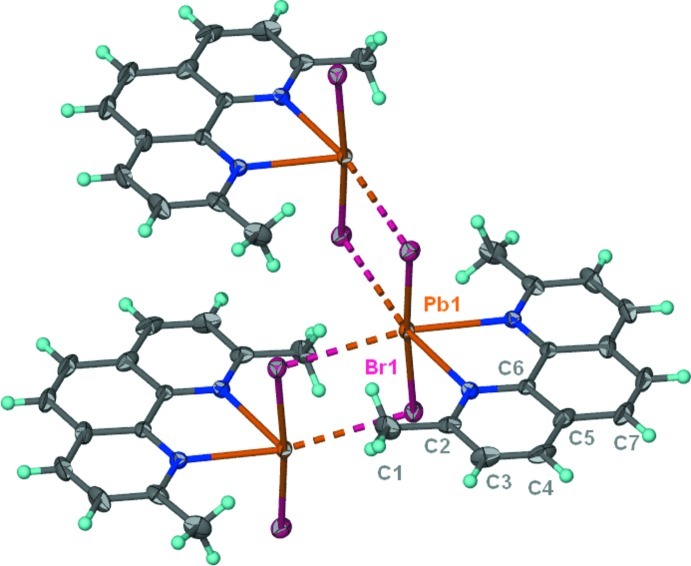
Anisotropic displacement ellipsoid plot (Barbour, 2001) of PbBr2(C14H12N2) at the 70% probability level; hydrogen atoms are drawn as spheres of arbitrary radius; symmetry-related atoms are not labeled. Adjacent molecules are linked by a weak Pb···Br bond, which is denoted as a dashed line.

## supplementary crystallographic information

### Comment

The *N*-heterocycle chelated Pb^II^ atom in PbBr_2_(C_14_H_12_N_2_) exists in a slightly distorted octahedral geometry with Pb···Br distances of 2.9562 (5)Å and 3.2594 (5)Å. The result are zigzag chains running along the *c*-axis of the monoclinic unit cell. The Pb centre lies on a twofold rotation axis. The lack of stereochemical activity can be attributed to crowding from the methyl substituents of the *N*-heterocycle (Bowmaker *et al.*, 1996).

### Experimental

Lead(II) bromide (0.37 g, 1 mmol) and 2,9-dimethyl-1,10-phenanthroline (1/5, 1 mmol) were loaded in a convection tube; the tube was filled with methanol and kept at 333 K. Colorless crystals were collected from the side arm after several days.

### Refinement

H-atoms were placed in calculated positions [C–H 0.95 to 0.98 Å, *U*_iso_(H) 1.2 to 1.5*U*_eq_(C)] and were included in the refinement in the riding model approximation.

The final difference Fourier map had a peak at 0.82 Å and a hole at 1.01 Å from Pb1.

### Crystal data

**Table:**

[PbBr_2_(C_14_H_12_N_2_)]	*F*(000) = 1048
*M**_r_* = 575.27	*D*_x_ = 2.546 Mg m^−^^3^
Monoclinic, *C*2/*c*	Mo *K*α radiation, λ = 0.71073 Å
Hall symbol: -C 2yc	Cell parameters from 2739 reflections
*a* = 18.3852 (13) Å	θ = 2.4–27.5°
*b* = 11.8312 (5) Å	µ = 16.55 mm^−^^1^
*c* = 7.4609 (5) Å	*T* = 100 K
β = 112.346 (8)°	Prism, colorless
*V* = 1501.02 (16) Å^3^	0.15 × 0.15 × 0.05 mm
*Z* = 4	

### Data collection

**Table:**

Agilent SuperNova Dual diffractometer with an Atlas detector	1734 independent reflections
Radiation source: SuperNova (Mo) X-ray Source	1620 reflections with *I* > 2σ(*I*)
Mirror monochromator	*R*_int_ = 0.032
Detector resolution: 10.4041 pixels mm^-1^	θ_max_ = 27.6°, θ_min_ = 2.4°
ω scan	*h* = −22→23
Absorption correction: multi-scan (*CrysAlis PRO*; Agilent, 2012)	*k* = −15→11
*T*_min_ = 0.190, *T*_max_ = 0.492	*l* = −8→9
4947 measured reflections	

### Refinement

**Table:**

Refinement on *F*^2^	Primary atom site location: structure-invariant direct methods
Least-squares matrix: full	Secondary atom site location: difference Fourier map
*R*[*F*^2^ > 2σ(*F*^2^)] = 0.026	Hydrogen site location: inferred from neighbouring sites
*wR*(*F*^2^) = 0.058	H-atom parameters constrained
*S* = 1.01	*w* = 1/[σ^2^(*F*_o_^2^) + (0.0298*P*)^2^] where *P* = (*F*_o_^2^ + 2*F*_c_^2^)/3
1734 reflections	(Δ/σ)_max_ = 0.001
88 parameters	Δρ_max_ = 1.43 e Å^−^^3^
0 restraints	Δρ_min_ = −1.45 e Å^−^^3^

### Fractional atomic coordinates and isotropic or equivalent isotropic displacement parameters (Å^2^)

**Table:**

	*x*	*y*	*z*	*U*_iso_*/*U*_eq_	
Pb1	0.5000	0.399831 (16)	0.2500	0.01318 (9)	
Br1	0.40287 (3)	0.38883 (3)	0.48715 (6)	0.01909 (12)	
N1	0.5735 (2)	0.2162 (3)	0.3998 (5)	0.0138 (7)	
C1	0.6822 (3)	0.3265 (4)	0.6184 (6)	0.0261 (11)	
H1A	0.6768	0.3765	0.5092	0.039*	
H1B	0.7381	0.3151	0.6972	0.039*	
H1C	0.6563	0.3610	0.6980	0.039*	
C2	0.6450 (3)	0.2162 (4)	0.5439 (6)	0.0183 (9)	
C3	0.6839 (3)	0.1139 (4)	0.6216 (7)	0.0241 (11)	
H3	0.7345	0.1152	0.7230	0.029*	
C4	0.6485 (3)	0.0134 (4)	0.5503 (6)	0.0250 (11)	
H4	0.6743	−0.0554	0.6030	0.030*	
C5	0.5748 (3)	0.0111 (3)	0.4008 (6)	0.0206 (10)	
C6	0.5374 (3)	0.1159 (3)	0.3270 (6)	0.0141 (9)	
C7	0.5352 (4)	−0.0932 (3)	0.3218 (7)	0.0257 (12)	
H7	0.5598	−0.1631	0.3730	0.031*	

### Atomic displacement parameters (Å^2^)

**Table:**

	*U*^11^	*U*^22^	*U*^33^	*U*^12^	*U*^13^	*U*^23^
Pb1	0.01465 (15)	0.01085 (12)	0.01436 (13)	0.000	0.00588 (10)	0.000
Br1	0.0217 (3)	0.0169 (2)	0.0212 (2)	−0.00258 (17)	0.0109 (2)	−0.00127 (15)
N1	0.014 (2)	0.0143 (16)	0.0136 (16)	0.0002 (15)	0.0052 (15)	−0.0005 (13)
C1	0.016 (3)	0.035 (3)	0.021 (2)	−0.004 (2)	0.001 (2)	−0.0017 (19)
C2	0.016 (3)	0.026 (2)	0.0156 (19)	−0.0006 (19)	0.0089 (19)	0.0041 (17)
C3	0.014 (3)	0.034 (3)	0.023 (2)	0.009 (2)	0.005 (2)	0.0105 (18)
C4	0.032 (3)	0.024 (2)	0.024 (2)	0.013 (2)	0.017 (2)	0.0115 (19)
C5	0.028 (3)	0.017 (2)	0.024 (2)	0.0061 (19)	0.018 (2)	0.0055 (17)
C6	0.020 (3)	0.0131 (19)	0.0137 (19)	0.0009 (16)	0.011 (2)	0.0008 (14)
C7	0.045 (4)	0.011 (2)	0.032 (3)	0.0033 (19)	0.028 (3)	0.0029 (16)

### Geometric parameters (Å, º)

**Table:**

Pb1—N1^i^	2.578 (3)	C2—C3	1.413 (6)
Pb1—N1	2.578 (3)	C3—C4	1.362 (7)
Pb1—Br1^i^	2.9562 (5)	C3—H3	0.9500
Pb1—Br1	2.9562 (5)	C4—C5	1.390 (7)
Pb1—Br1^ii^	3.2594 (5)	C4—H4	0.9500
N1—C2	1.345 (6)	C5—C6	1.423 (5)
N1—C6	1.368 (5)	C5—C7	1.440 (6)
C1—C2	1.481 (6)	C6—C6^i^	1.417 (9)
C1—H1A	0.9800	C7—C7^i^	1.330 (12)
C1—H1B	0.9800	C7—H7	0.9500
C1—H1C	0.9800		
			
N1^i^—Pb1—N1	65.15 (16)	N1—C2—C3	121.1 (4)
N1^i^—Pb1—Br1^i^	92.27 (7)	N1—C2—C1	118.1 (4)
N1—Pb1—Br1^i^	83.46 (7)	C3—C2—C1	120.8 (4)
N1^i^—Pb1—Br1	83.46 (7)	C4—C3—C2	119.6 (5)
N1—Pb1—Br1	92.27 (7)	C4—C3—H3	120.2
Br1^i^—Pb1—Br1	174.955 (16)	C2—C3—H3	120.2
N1^i^—Pb1—Br1^ii^	169.82 (7)	C3—C4—C5	120.4 (4)
N1—Pb1—Br1^ii^	107.98 (8)	C3—C4—H4	119.8
Br1^i^—Pb1—Br1^ii^	94.383 (13)	C5—C4—H4	119.8
Br1—Pb1—Br1^ii^	89.490 (13)	C4—C5—C6	118.3 (4)
C2—N1—C6	119.7 (4)	C4—C5—C7	122.1 (4)
C2—N1—Pb1	122.6 (3)	C6—C5—C7	119.6 (5)
C6—N1—Pb1	117.6 (3)	N1—C6—C6^i^	119.8 (2)
C2—C1—H1A	109.5	N1—C6—C5	120.8 (4)
C2—C1—H1B	109.5	C6^i^—C6—C5	119.4 (3)
H1A—C1—H1B	109.5	C7^i^—C7—C5	121.0 (3)
C2—C1—H1C	109.5	C7^i^—C7—H7	119.5
H1A—C1—H1C	109.5	C5—C7—H7	119.5
H1B—C1—H1C	109.5		
			
N1^i^—Pb1—N1—C2	179.5 (4)	C2—C3—C4—C5	0.5 (7)
Br1^i^—Pb1—N1—C2	83.9 (3)	C3—C4—C5—C6	−0.8 (7)
Br1—Pb1—N1—C2	−98.8 (3)	C3—C4—C5—C7	−179.8 (4)
Br1^ii^—Pb1—N1—C2	−8.6 (3)	C2—N1—C6—C6^i^	−179.0 (4)
N1^i^—Pb1—N1—C6	−0.2 (2)	Pb1—N1—C6—C6^i^	0.7 (6)
Br1^i^—Pb1—N1—C6	−95.8 (3)	C2—N1—C6—C5	−0.5 (6)
Br1—Pb1—N1—C6	81.5 (3)	Pb1—N1—C6—C5	179.2 (3)
Br1^ii^—Pb1—N1—C6	171.7 (3)	C4—C5—C6—N1	0.8 (6)
C6—N1—C2—C3	0.2 (6)	C7—C5—C6—N1	179.8 (4)
Pb1—N1—C2—C3	−179.5 (3)	C4—C5—C6—C6^i^	179.2 (5)
C6—N1—C2—C1	179.8 (4)	C7—C5—C6—C6^i^	−1.7 (7)
Pb1—N1—C2—C1	0.1 (5)	C4—C5—C7—C7^i^	−179.7 (5)
N1—C2—C3—C4	−0.3 (7)	C6—C5—C7—C7^i^	1.3 (8)
C1—C2—C3—C4	−179.8 (4)		

Symmetry codes: (i) −*x*+1, *y*, −*z*+1/2; (ii) −*x*+1, −*y*+1, −*z*+1.
